# Lesbian, Gay, Bisexual, Transgender, Queer, Intersex, Asexual, and Other Minoritized Gender and Sexual Identities–Adapted Telehealth Intensive Outpatient Program for Youth and Young Adults: Subgroup Analysis of Acuity and Improvement Following Treatment

**DOI:** 10.2196/45796

**Published:** 2023-04-21

**Authors:** Katie R Berry, Kate Gliske, Clare Schmidt, Ley David Elliette Cray, Michael Killian, Caroline Fenkel

**Affiliations:** 1 Charlie Health, Inc Bozeman, MT United States; 2 College of Social Work Florida State University Tallahassee, FL United States

**Keywords:** lesbian, gay, bisexual, transgender, queer, intersex, asexual, and other minoritized gender and sexual identities, LGBTQIA+, youth, mental health, affirming health care, suicidal ideation, depression, nonsuicidal self-harm, NSSI

## Abstract

**Background:**

Lesbian, gay, bisexual, transgender, queer, intersex, asexual, and other minoritized gender and sexual identities (LGBTQIA+) youth have disproportionately high levels of depression, self-harm, and suicidal thoughts and behaviors. In addition, LGBTQIA+ youth frequently report lower levels of satisfaction or comfort with their health care providers because of stigmatization, which may prevent continuation of care, yet there is a lack of mental health treatment and outcome research addressing these disparities. However, there is some indication that LGBTQIA+ individuals feel more comfortable with web-based formats, indicating that telehealth services may be beneficial for this population.

**Objective:**

This program evaluation explored the effectiveness of a remote intensive outpatient program with a curriculum tailored specifically to LGBTQIA+ youth with high-acuity depression, anxiety, and suicidality. This study sought to understand baseline acuity differences between LGBTQIA+ and non-LGBTQIA+ youth and young adult patients and to determine if there were differences in clinically significant improvement by subtypes within the LGBTQIA+ population following participation in LGBTQIA+-specific programming.

**Methods:**

Data were collected from intake and discharge outcome surveys measuring depression, suicidality, and nonsuicidal self-injury (NSSI) in 878 patients who attended at least six sessions of a remote intensive outpatient program for youth and young adults. Of these 878 clients, 551 (62.8%) were identified as having at least one LGBTQIA+ identity; they participated in an LGBTQIA+-adapted program of the general curriculum.

**Results:**

LGBTQIA+ patients had more clinically severe intake for depression, NSSI, and suicidal ideation. Nonbinary clients had greater NSSI within the LGBTQIA+ sample at intake than their binary counterparts, and transgender clients had significantly higher depressive scores at intake than their nontransgender counterparts. LGBTQIA+ patients demonstrated improvements in all outcomes from intake to discharge. The Patient Health Questionnaire for Adolescents depression scores improved from 18.15 at intake to 10.83 at discharge, representing a 41.5% reduction in depressive symptoms. Overall, 50.5% (149/295) of the LGBTQIA+ youth who endorsed passive suicidal ideation at intake no longer reported it at discharge, 72.1% (160/222) who endorsed active suicidal ideation at intake no longer reported it at discharge, and 55.1% (109/198) of patients who met the criteria for clinical NSSI no longer met the criteria at discharge. In the subgroup analysis, transgender patients were still 2 times more likely to report clinical NSSI at discharge.

**Conclusions:**

This program evaluation found substantial differences in rates of depression, NSSI, and suicidal ideation between LGBTQIA+ clients compared with their non-LGBTQIA+ counterparts. In addition, this evaluation showed a considerable decrease in symptoms when clients attended LGBTQIA+-affirming care. The findings provide support for the role of LGBTQIA+-specific programming to meet the elevated mental health needs of these youth and that more research is needed to understand barriers that may negatively affect transgender clients, specifically.

## Introduction

### Background

Suicide is a leading cause of death in youth and young adults aged 10 to 24 years in the United States [1]. Between 2007 and 2020, the suicide rate steadily increased in the United States for this age group [2]. Within this age group, the risk for suicide is especially pronounced within certain minoritized communities: compared with heterosexual and cisgender youth, youth who identify as lesbian, gay, bisexual, transgender, queer, intersex, asexual, and other minoritized gender and sexual identities (LGBTQIA+) report higher levels of suicidal ideation and attempts; report higher rates of suicide risk factors, such as depression, substance use, and self-harm; and are more likely to disclose a past history of abuse, victimization, bullying, or trauma [3-6]. Despite disproportionately high levels of depression, self-harm, and suicidal thoughts and behaviors in LGBTQIA+ youth relative to the general youth population, there remains a lack of suicide-specific mental health treatment and research addressing this disparity [7]. Preliminary research indicates that LGBTQIA+ youth who participate in adapted treatments demonstrate considerable clinical improvement after treatment [8] and that LGBTQIA+ youth describe the treatment as helpful in addressing their life stresses [9]. Given the disparities and the importance of intervening in serious mental health issues in adolescence and young adulthood, there is a critical need for more research on the efficacy of general and population-specific programming for LGBTQIA+ youth [10].

Studies including LGBTQIA+ youth have consistently highlighted the heightened risk of suicide among this population relative to cisgender and heterosexual youth [11,12]. Given the diversity of gender and sexual identities within the LGBTQIA+ community, subpopulation differences within this group have been explored and yielded findings that substantiate the heterogeneity of risk within this community [13,14]. For example, Jadva et al [13] compared transgender and nontransgender youth within the LGBTQIA+ community and found that transgender youth were 4 times as likely to report self-harm, 3 times more likely to report suicidal ideation, and 2 and a half times as likely to report having attempted suicide. In 1 study of gender minority youth, sex assigned at birth was linked to a higher risk of mental health, self-harm, and sexual assault, and nonbinary youth had higher life satisfaction [14]. Thus, when designing mental health programming and evaluating mental health treatment for LGBTQIA+ youth, it is important to use an evaluative approach that allows the exploration of subgroup differences. However, individual organizations and practices may be limited in how nuanced an analysis they are able to perform, given that small subsample sizes often do not lend themselves to sufficiently powered analyses.

Despite the elevated risk, mental health treatment for LGBTQIA youth can lead to notable symptom improvement and increased well-being. Both treatments adapted for sexual and gender minority clients and treatments with no modifications have reported significant improvements in clinical outcomes [15]. Adapted treatments can address stigma-related stressors and processes that impact both health and health care experiences, such as anxious expectations of rejection or concealed sexual orientation [16]. LGBTQIA+ individuals often report lower satisfaction with their health care experiences and barriers that prevent the initiation and continuation of care [17-19]. Barriers may include a lack of LGBTQIA+-specific clinical competencies, discrimination, the fear of disclosing one’s gender identity or sexual orientation, financial burden, and a practitioner’s perceived or actual lack of willingness to discuss sexuality or gender identity [19-23]. These barriers may lead to decreased willingness to seek treatment and hinder the development of a therapeutic relationship, potentially reducing the effectiveness of treatment [19]. Adapted interventions may help address these processes and barriers, leading to stronger outcomes. To our knowledge, only 1 study has directly compared sexuality-tailored and gender minority–tailored interventions to an intervention without adaptations. This substance use intervention found that the tailored intervention led to greater reductions in substance use [24]. In 2 studies that adapted programming for LGBTQIA+ youth, youth had significantly reduced posttraumatic stress disorder symptoms [25], depression, and borderline symptoms after treatment [8]. Research on adults has similarly documented clinical improvement following adapted interventions [26,27].

Youth broadly face difficulty in finding a therapist, with shortages in mental health professionals prevalent globally [28,29]. This shortage is amplified for LGBTQIA+ youth, as there are even fewer mental health practitioners with expertise and training to specifically address the challenges unique to the LGBTQIA+ community [30]. For instance, among transgender individuals, having a transgender-inclusive provider was associated with decreased rates of depression and suicidality, indicating that one key aspect of effective mental health programming for this population may be improving the LGBTQIA+ competency of mental health providers [31]. Finally, the increased difficulty in accessing quality care is particularly salient for youth living in rural settings [32,33]. This may be exacerbated for LGBTQIA+ youth, as they may not feel safe discussing their gender identity or sexuality with their parents or they may fear that mental health professionals could *out* them, try to *cure* their gender identity or sexuality, or turn them away for being LGBTQIA+ [30].

Given the disproportionately high rates of depression, self-harm, and suicidality experienced by LGBTQIA+ youth, there is a growing need for high-quality and accessible mental health services tailored specifically to this population [9]. Even before the COVID-19 pandemic, telehealth was assessed as a way to ameliorate the local shortages of mental health professionals for both youth and adults and as a way to increase access in more rural areas [34-37]. Patients can access telehealth services by phone or computer from anywhere on the internet, removing barriers, such as not having transportation or the time spent commuting, and broadening the scope and availability of services [36,38]. Telehealth treatment may be uniquely beneficial to LGBTQIA+ youth, as this population already has a high use of technology to connect with web-based communities, build support and resilience, and seek resources and information [39,40]. LGBTQIA+ youth also report feeling safer in web-based communities than in offline forums [41,42]. A review of early research on telepsychiatry and LGBT treatment gaps suggests that telepsychiatry can bypass some of the stigma related to seeking treatment and that it allows clients more opportunities to seek a culturally competent therapist without being restricted by geographic proximity [43]. Digital health interventions have high acceptability and low attrition rates among LGBTQIA+ young people [44].

### Objectives

The primary objective of this program evaluation was to explore the effectiveness of a curriculum tailored specifically to LGBTQIA+ youth in a remote intensive outpatient program (IOP). This evaluation is part of an ongoing routine outcome monitoring system, primarily aimed at identifying opportunities for quality improvement in care. Given the lack of research on LGBTQIA+ youth seeking mental health services, this study sought to answer the following questions:

- Are LGBTQIA+ youth and young adult patients more clinically severe at intake and discharge relative to non-LGBTQIA+ patients?
- Are LGBTQIA+ youth and young adult patients experiencing clinically significant changes from intake to discharge in LGBTQIA+-specific programming?
- Are there subgroup differences within the LGBTQIA+ community in terms of clinical improvement and acuity at discharge?

## Methods

### Overview

Charlie Health is a remote mental and behavioral health IOP provider serving adolescents and young adults (aged 11-28 years). Charlie Health regularly collects and analyzes patient-reported outcomes data using validated measures to ensure the quality of care and to meet the requirements of stakeholders and accrediting bodies. To increase treatment responsiveness, Charlie Health engages in ongoing program evaluations to identify opportunities for quality improvements. This program evaluation is part of a larger effort to assess what works for youth with mental health needs that necessitate IOP.

### Ethics Approval

This program evaluation research was reviewed by the Florida State University Institutional Review Board, which deemed this investigation “nonhuman subjects research,” given its primary purpose of program evaluation and quality improvement (STUDY00003364).

### Sample and Program Characteristics

#### Overview

The sample included patients discharged from Charlie Health services between December 20, 2020, and May 31, 2022. Inclusion criteria were limited to (1) patient cases with both an intake and discharge survey and (2) patients with 6 or more IOP sessions. The latter criterion was determined based on dose-response research, which indicates that at least 18 hours of sessions (approximately 6 IOP sessions) are necessary to observe clinically significant changes in acute mental health symptoms [45-47]. The sample comprised patients who successfully completed treatment and those who were discharged for one of several reasons (ie, disengagement, insurance denial, or referral to a higher or lower level of care). The resulting sample included 878 patients and a subsample of 551 patients identified as having at least one LGBTQIA+ identity.

In general, the patient population of Charlie Health is characterized by high clinical acuity, presenting with one or more co-occurring mental and behavioral health issues that require intensive services beyond what can be delivered in less intense community-based care. Patients typically present with variable historical exposure to treatment (they may be stepping down from a higher level of care, up from a lower level of care, or initiating treatment for the first time). Furthermore, patients come from variable socioeconomic backgrounds as the program accepts both public and private insurances.

The Charlie Health IOP program provides 9 hours of group sessions with an optional 1-hour individual or 1-hour family session per week, determined by need and patient (or caregiver or family) willingness to participate. The average length of treatment stay is 10 to 12 weeks. Patients are assigned an IOP group track based on their presenting issues (ie, depression and substance use) and identities (ie, gender, age, and sexual orientation). One of the advantages of being a national provider is the ability to assign patients to groups that are both identity-affirming and presenting issue focused (ie, trauma-focused cognitive behavioral therapy for older LGBTQIA+ patients). Group sessions are offered at various times throughout the day to work with patients’ schedules, thereby increasing the accessibility of services. Group sessions are 3 hours long, broken out into three 50-minute sessions that included evidence-based skill building interventions (ie, dialectical behavior therapy [DBT] and cognitive behavioral therapy), general therapeutic processing, and experiential therapy (ie, art, music, and journaling). In addition to individual, family, and group sessions, Charlie Health has a robust family support program that provides psychoeducational and support groups to caregivers and other treatment-involved loved ones.

#### LGBTQIA+ Programming

Charlie Health offers an LGBTQIA+-specific adaptation of the general curriculum that is accessible to LGBTQIA+ youth across the United States, who may be struggling with depression, self-harm, and suicidality. The LGBTQIA+ IOP program was the first web-based program designed to address acute and severe depression, self-harm, and suicidality in LGBTQIA+ adolescents and young adults.

The Charlie Health LGBTQIA+ remote IOP curriculum uses a clinical model that centers *affirming care*, including best practices for LGBTQIA+ care that are in alignment with the Diagnostic and Statistical Manual of Mental Health Disorders [48] and guidelines and standards proposed by the American Medical Association [49], American Psychological Association [50], and the World Professional Association for Transgender Health [51]. The program emphasizes a minority stress framework for the delivery of care while also depathologizing LGBTQIA+ identities, approaching such identities as natural variations in human experience and self-conception rather than as conditions to be cured.

Facilitators of these groups receive regular training and enrichment opportunities on clinical engagement with LGBTQIA+ populations and have access to weekly consultation hours led by subject matter experts through which they can consult on cases; discuss best and promising practices; and continue their education on LGBTQIA+ care, concepts, and communities. Along with other topics, trainings center intersectional approaches that emphasize the inseparability of considerations of patient gender and sexual identity from other aspects of identity (eg, race and ethnicity) [51-53].

As permitted by training and availability, LGBTQIA+ groups are primarily facilitated by facilitators who claim at least one LGBTQIA+ identity. Curricula used throughout all patient groups adapt Charlie Health’s general curriculum with additional questions that explicitly and intentionally foreground patient reflections on and affirmations of LGBTQIA+ identity and community.

Research confirms that family engagement and support are predictive of success in achieving positive results when working with LGBTQIA+ populations [54-56]. To this end, loved ones of Charlie Health patients have access to a weekly support group specifically for parents and caregivers of LGBTQIA+ youth (eg, information on gender identity and expression).

### Data Collection Procedures

Data were collected during the patients’ first (intake) and last (discharge) IOP sessions. When patients entered the web-based group room, they were sent to a survey room, where a staff member provided them with a Qualtrics (Qualtrics) [57] link to complete either the intake or discharge survey. Patients who missed their last IOP session were emailed the discharge survey with a small incentive for completion. All patient data were downloaded and deidentified for program evaluation.

### Measures

Patient demographic data were collected at intake (age) and discharge (sexual orientation and gender identity). Patients were asked to select the sexual orientation that they most identified with from response options that included asexual or graysexuality, bisexual, pansexual, gay, heterosexual or straight, lesbian, queer, and questioning. Similarly, patients were asked to select the gender identity that they most identified with from the following options: gender fluid, gender neutral, man, woman, gender questioning, genderqueer or nonconforming, nonbinary, and “other.” Finally, the patients were asked if they identified as transgender (yes or no). The intake and discharge surveys also included a battery of clinical assessments to assess pre- and posttherapeutic changes, including measures of depression, anxiety, suicidal ideation, and self-harm.

### Outcome Variables

#### Depression

The Patient Health Questionnaire for Adolescents (PHQ-A) is an age-adapted measure intended to screen and capture changes in depression severity among adolescents [58]. The PHQ-A, the 9-item version of the Patient Health Questionnaire (PHQ), has been well established as a valid and sensitive tool for screening and detecting changes in depression [59]. PHQ-A has a range of 0 to 27. Interpretation of scores on the PHQ-A is as follows: 0 to 4 is “minimal,” 5 to 9 is “mild,” 10 to 14 is “moderate,” 15 to 19 is “moderately severe,” and 20 to 27 is “severe.” The clinical cutoff for the PHQ-A is a score ≥10. A score change of at least 5 was considered a “clinically significant” change [59]. In the current sample, the PHQ-A demonstrated good reliability at the pretest (a=.91) and posttest (a=.91).

#### Suicide Risk

The risk of suicide was measured using the Ask Suicide-Screening Questionnaire (ASQ). The ASQ asks a set of 4 dichotomous questions (yes or no) about passive and active suicidal ideation and a history of attempts. A positive response to any of these questions resulted in a positive screening status for suicide risk. In a large National Institute of Mental Health study using a pediatric sample, the ASQ was found to have excellent sensitivity (96.9%, 95% CI 91.3-99.4) and specificity (87.6%, 95% CI 84-90.5) [60].

#### Clinical Nonsuicidal Self-injury

The Alexian Brothers Assessment of Suicidal Ideation (ABASI) was used to assess clinical nonsuicidal self-injury (NSSI) [61]. The ABASI lists 21 different types of self-injury (SI), asking respondents to indicate how many days in the past 30 they had engaged in each subtype. Endorsement of 5 or more days on any type of SI behavior is interpreted as “meeting criteria” for clinical NSSI. The ABASI has demonstrated adequate internal consistency and test-retest reliability among adolescents and young adults [61,62].

### Identity Variable

#### Identifying LGBTQIA+ Patients

Patients who identified as nonheterosexual, gender-diverse (not as a man or woman), or transgender were categorized as “LGBTQIA+” patients; all patients who identified as heterosexual and cisgender were classified as “non-LGBTQIA+.”

#### Data Preparation

Several new variables were computed and recorded to conduct the main analyses included in this evaluation.

#### Suicidal Risk

Suicidal risk was assessed at intake using 3 variables: passive suicidal ideation, active suicidal ideation, and history of suicide attempts. Passive suicidal ideation was operationalized by summing questions 1 and 2 on the ASQ and recoding the sum scores into a dichotomous screening variable, where a score of 0 is “negative” and a score of 1 or 2 is “positive.” Active suicidal ideation (ASQ3) and history of attempts (ASQ4) were treated as separate dichotomous variables, where 0 was “negative” and 1 was “positive.” A total of 2 change variables were calculated for passive and active suicidal ideation.

#### Clinical NSSI

To determine whether patients met the criteria for clinical NSSI, all 21 ABASI subtypes were recoded into dichotomous variables as follows: 0 (<5 days reported) and 1 (≥5 days reported). The sum of these variables was calculated, with a total range of 0 to 21. Cases with a sum score of “0” were labeled as “did not meet criteria” and those with a score of ≥1 were labeled as “met criteria” for clinical NSSI.

#### Gender and Sexual Identity Subgroups

To explore subgroup differences within the LGBTQIA+ patient community, the identity variables were recoded. For the first set of clinical acuity at intake comparisons between LGBTQIA+ and non-LGBTQIA+ patients, sexual orientation was collapsed into the 4 largest subgroups: heterosexual, pansexual, bisexual, and “other” (the former category combining all categorical response options that comprised <10% of the sample). Similarly, gender was collapsed into a 3-level categorical variable: man, woman, and nonbinary. Furthermore, to assess differences in sexual orientation and gender subtypes within the LGBTQIA+ sample at intake and in changes over time from intake to discharge, sexual orientation was recoded into 2 separate variables: bisexual (1=bisexual and 0=nonbisexual) and pansexual (1=pansexual and 0=nonpansexual). Other sexual orientation groups were excluded from the current analyses because of low data coverage (~10% of the sample within the LGBTQIA+ population). For analyses comparing differences within the LGBTQIA+ sample, the gender variable was recoded as 0=binary and 1=nonbinary.

### Data Analysis Strategy

#### Overview

To assess changes in clinical symptoms, a series of paired samples 2-tailed *t* tests, McNemar tests, and logistic regression were used. Given the dearth of research on clinical samples from LGBTQIA+ youth, significant differences in clinical severity between LGBTQIA+ and non-LGBTQIA+ patients are presented. Analyses of clinical improvement were performed only on LGBTQIA+ patients and between subgroups within this population, as the clinical program designed for this population is an adapted version of the general curriculum, thus obviating the utility of a clinical improvement comparison between LGBTQIA+ and non-LGBTQIA+ patients. When assessing clinical outcomes and between-group differences, intake scores for each measure were included in the general linear model (ie, repeated measures ANOVA or logistic regression) to estimate less biased between-group differences. A Bonferroni correction was used to control for inflation of type 1 error given the examination of clinical change over 4 different patient self-report measures (ie, depression, both passive and active suicidality, and NSSI scores). As such, the significance level for this portion of the analyses was ⍺=.0125 (.05/4).

#### Depression

Independent samples *t* tests were used to assess significant differences between LGBTQIA+ and non-LGBTQIA+ patients at intake. Repeated measures ANOVA was also used to explore changes across the whole sample of LGBTQIA+ patients and by transgender and gender identity (binary vs nonbinary) subgroups.

#### Suicide Risk

Chi-square analyses were used to assess significant differences between LGBTQIA+ and non-LGBTQIA+ patients on passive (ASQ1 through 2) and active suicidal thoughts (ASQ3) and history of suicide attempts (ASQ4). McNemar test was used to assess changes in passive and active suicidal thoughts across the sample of LGBTQIA+ patients who screened positive for SI at intake. Binary logistic regression was used to assess the subgroup differences in the change from intake to discharge.

#### Clinical NSSI

Chi-square analysis was used to explore the differences in NSSI at intake between LGBTQIA+ and non-LGBTQIA+ patients. Significant changes in the NSSI criteria from intake to discharge were assessed using the McNemar test. Binary logistic regression was used to assess the subgroup differences in the change from intake to discharge.

### Missing Data

Given the nature of these secondary data analyses as part of an ongoing quality improvement and assurance initiative, missing data affected 2 aspects of the evaluation. First, because gender and sexual orientation were collected as part of the discharge survey, we were unable to compare the demographic characteristics of patients who left treatment before the engagement threshold or who did not complete the discharge survey with those who were ultimately included in the current analysis. Second, there were missing data on some demographic and clinical variables, resulting in variable subsample sizes across the tests. This is a consequence of not “forcing” survey responses, wherein patients are encouraged to complete the surveys fully but have the option to skip over questions they do not wish to answer.

## Results

### Patient Demographic Characteristics

During the study period, 1766 individuals were discharged from programming, of which 1275 (72.22%) met the engagement criteria of attending at least 6 IOP sessions. Among clients who did not meet the inclusion criteria based on the number of sessions attended, 73.7% (362/491) were discharged within the first week of treatment and had attended ≤3 IOP sessions. Of the 1275 clients who met the engagement threshold, 397 (31.14%) did not complete the discharge survey, resulting in 878 (68.86%) clients included in the final sample.

The demographics of LGBTQIA+ and non-LGBTQIA+ samples are presented in Table 1. Compared with non-LGBTQIA+ patients, LGBTQIA+ patients were significantly younger (mean 16.44, SD 3.80 vs mean 17.01, SD 4.03 years; t_871_=2.11; *P=*.04; Cohen *d=*0.14).

**Table 1:** LGBTQIA+^a^ and non-LGBTQIA+ patient demographic characteristics.

			Non-LGBTQIA+ (n=327)		LGBTQIA+ (n=551)
Age (years), mean (SD)			17.01 (4.03)		16.44 (3.80)
**Gender, n (%)**					
	Man	145 (44.6)		105 (19.1)	
	Woman	182 (55.7)		251 (45.6)	
	Gender fluid	0 (0)		51 (9.3)	
	Gender neutral	0 (0)		10 (1.8)	
	Gender questioning	0 (0)		20 (3.6)	
	Genderqueer	0 (0)		6 (1.1)	
	Gender conforming	0 (0)		17 (3.1)	
	Nonbinary	0 (0)		90 (16.3)	
	Missing	0 (0)		1 (0.2)	
**Transgender, n (%)**					
	Not transgender	301 (92)		387 (70.2)	
	Transgender	0 (0)		161 (29.2)	
	Missing	26 (8)		3 (0.5)	
**Sexual orientation, n (%)**					
	Asexual or graysexual	0 (0)		39 (7.1)	
	Bisexual	0 (0)		189 (34.3)	
	Pansexual	0 (0)		145 (26.3)	
	Gay	0 (0)		27 (4.9)	
	Heterosexual or straight	289 (88.4)		7 (1.3)	
	Lesbian	0 (0)		41 (9.3)	
	Queer	0 (0)		51 (9.3)	
	Questioning	38 (11.6)		52 (9.4)	
**Number of intersectional identities, n (%)**					
	1 LGBTQIA+ identity	0 (0)		285 (51.7)	
	2 LGBTQIA+ identities	0 (0)		184 (33.4)	
	3 LGBTQIA+ identities	0 (0)		82 (14.9)	

^a^LGBTQIA+: lesbian, gay, bisexual, transgender, queer, intersex, asexual, and other minoritized gender and sexual identities.

### Baseline Clinical Characteristics

#### Overview

A series of *χ*^2^ analyses and an independent samples *t* test were used to assess clinical differences at baseline between LGBTQIA+ (551/878, 62.8%) and non-LGBTQIA+ patients (327/878, 37.2%) and between groups within the LGBTQIA+ community. In almost all domains, patients with an LGBTQIA+ identity were significantly more clinically severe at baseline than their non-LGBTQIA+ peers.

#### Suicide Risk

##### Overview

Compared with non-LGBTQIA+ patients at treatment intake, LGBTQIA+ patients had a significantly greater proportion of positive screens for passive suicidal ideation at treatment intake (305/448, 68.1% vs 134/258, 51.9%; *χ*^2^_1_=18.1, n=706, *P<*.001). LGBTQIA+ patients were significantly more likely to report active SI compared with non-LGBTQIA+ patients (229/503, 45.5% vs 86/279, 30.8%; *χ*^2^_1_=16.1, n=782, *P<*.001). LGBTQIA+ patients were also significantly more likely to report a history of suicide attempts compared with non-LGBTQIA+ patients (242/447, 54.1% vs 112/258, 43.4%; *χ*^2^_1_=7.5, n=705, *P=*.006; Table 2).

**Table 2:** Baseline differences in suicide risk by patient group (LGBTQIA+^a^ vs non-LGBTQIA+).

				Patient group					Test				*P* value			Effect size	
				Non-LGBTQIA+		LGBTQIA+											
**Passive SI^b^ (non-LGBTQIA+: n=258; LGBTQIA: n=448), n (%)**										*χ*^2^_1_=18.1				<.001			Φ=0.16
	Negative		124 (48.1)		143 (31.9)												
	Positive		134 (51.9)		305 (68.1)												
**Active SI (non-LGBTQIA+: n=279; LGBTQIA: n=503), n (%)**										*χ*^2^_1_=16.1				<.001			Φ=0.14
	Negative		193(69.2)		274 (54.5)												
	Positive		86 (30.8)		229 (45.5)												
**History of attempt (non-LGBTQIA+: n=258; LGBTQIA: n=447), n (%)**										*χ*^2^_1_=7.5				.003			Φ=0.10
	Negative		146 (56.6)		205 (45.9)												
	Positive		112 (43.4)		242 (54.1)												
**Clinical NSSI^c^ (non-LGBTQIA+: n=206; LGBTQIA: n=388), n (%)**										*χ*^2^_1_=19.8				<.001			Φ=0.18
	Did not meet criteria		136 (66)		182 (46.9)												
	Met criteria		70 (34)		206 (53.1)												
Depression, mean (SD)				11.71 (7.76)		15.31 (7.16)		t_628.92_*=*6.62				<.001			Cohen *d=*−0.48		

^a^LGBTQIA+: lesbian, gay, bisexual, transgender, queer, intersex, asexual, and other minoritized gender and sexual identities.

^b^SI: self-injury.

^c^NSSI: nonsuicidal self-injury.

##### Within LGBTQIA+ Patients

Among LGBTQIA+ patients, no statistically significant differences were found in suicidal ideation. No differences were detected in passive suicidal ideation among transgender and nontransgender patients (100/135, 74.1% vs 219/335, 65.4%; χ 2 1=3.34, n=470, *P*=.07), bisexual and nonbisexual patients (108/154, 70.1% vs 212/318, 66.7%; χ 2 1=.57, n=472, *P*=.45), pansexual and nonpansexual patients (92/127, 72.4% vs 228/345, 66.1%; χ 2 1=1.72, n=472, *P*=.19), and nonbinary and binary patients (122/170, 71.8% vs 197/301, 65.4%; χ 2 1=1.98, n=471, *P*=.16).

No differences were detected in active suicidal ideation among transgender and nontransgender patients (67/151, 44.4% vs 168/374, 44.9%; χ 2 1=.01, n=525, *P*=.91), bisexual and nonbisexual patients (80/179, 44.7% vs 156/348, 44.8%; χ 2 1=.001, n=527, *P*=.98), pansexual and nonpansexual patients (67/143, 46.9% vs 169/384, 44.0%; χ 2 1=.34, n=527, *P*=.56), and nonbinary and binary patients (85/188, 45.2% vs 150/338, 44.4%; χ 2 1=.03, n=526, *P*=.85).

No differences were detected in history of attempts among transgender and nontransgender patients (67/134, 50.0% vs 185/335, 55.2%; χ 2 1=1.05, n=469, *P*=.31), bisexual and nonbisexual patients (87/153, 56.9% vs 166/318, 52.2%; χ 2 1=.90, n=471, *P*=.34), pansexual and nonpansexual patients (73/127, 57.5% vs 180/344, 52.3%; χ 2 1=.99, n=471, *P*=.32), and nonbinary and binary patients (94/170, 55.3% vs 158/300, 52.7%; χ 2 1=.30, n=470, *P*=.58).

#### Clinical NSSI

##### Overview

LGBTQIA+ patients were significantly more likely to meet the criteria for clinical NSSI compared with non-LGBTQIA+ patients at intake (206/338, 53.1% vs 70/206, 34%; *χ*^2^_1_=19.8, n=594, *P*<.001).

##### Within LGBTQIA+ Patients

Significant differences were not found between transgender and nontransgender patients (65/106, 61.3% vs 140/280, 50%; *χ*^2^_1_=4.0, n=386, *P=*.051) nor were they detected between bisexual and nonbisexual patients (63/135, 46.7% vs 143/253, 56.5%; *χ*^2^_1_=3.4, n=388, *P=*.06), However, nonbinary patients were significantly more likely to meet NSSI criteria, compared with binary patients at intake (85/138, 61.6% vs 121/250, 48.4%; *χ*^2^_1_=6.2, n=388, *P=*.02), and pansexual patients were significantly more likely to meet NSSI criteria relative to nonpansexual patients (70/112, 62.5% vs 136/276, 49.3%; *χ*^2^_1_=5.6, n=388, *P=*.02).

#### Depression Severity

##### Overview

The results of the independent samples *t* test indicate that patients who identified as having at least 1 LGBTQIA+ identity had significantly worse depression (mean 15.31, SD 7.16) compared with non-LGBTQIA+ patients (mean 11.71, SD 7.76) at intake (t_603.89_=6.64; *P*<.001).

##### Within LGBTQIA+ Patients

No significant differences were found between nonbinary (mean 15.93, SD 6.77) and binary patients (mean 14.95, SD 7.36) in baseline depressive symptoms at intake (t_516_=1.51; *P*=.12). Similarly, no significant differences were detected between bisexual (mean 15.69, SD 6.77) and nonbisexual patients (mean 15.10, SD 7.36; t_517_=−0.89; *P*=.37) or between pansexual (mean 15.19, SD 7.11) and nonpansexual patients (mean 15.35, SD 7.19; t_517_=0.22; *P*=.82). Transgender patients scored significantly higher (mean 16.32, SD 6.59) on baseline depression compared with nontransgender patients (mean 14.94, SD 7.36; t_291.77_=1.97; *P*=.04).

### Clinical Improvement

#### Overview

A series of analyses were performed to compare clinical improvement across LGBTQIA+ patients and between sexual and gender subgroups.

#### Depression Change

##### Overview

Among LGBTQIA+ patients who met the criteria for clinical depression at intake (PHQ-A score >10), the paired samples *t* test on change indicated a significant change of 7.69 from intake to discharge (*P<*.001). On average, clients in the sample reported a 41.5% reduction in depressive symptoms (Table 3).

**Table 3:** Change over time on depression, suicidal ideation, and clinical nonsuicidal self-injury (SI).

Variable			Intake		Discharge		Test		*P* value		Effect size
Depression, mean (SD)			18.51 (4.69)		10.83 (6.76)		t_392_=20.27		<.001		Cohen *d*=1.04
**Passive SI (n=432), n (%)**							*χ*^2^_1_=50.3		<.001		
	Yes	295 (68.3)		165 (38.2)						Φ=0.34	
	No	137 (31.7)		267 (61.8)							
**Active SI (n=486), n (%)**							*χ*^2^_1_=25.3		<.001		Φ=0.23
	Yes	222 (45.7)		89 (18.3)							
	No	264 (54.3)		397 (81.7)							
**Nonsuicidal SI (n=366), n (%)**							*χ*^2^_1_=26.2		<.001		Φ=0.27
	Yes	198 (54.1)		122 (33.3)							
	No	168 (45.9)		244 (66.7)							

##### Depression Change Between Gender Subgroups

The findings of the repeated measures ANOVA indicated a significant change in PHQ scores over time (*F*_1,488_=218.85; *P<*.001). Although transgender identity was a significant between-subjects predictor (*F*_1,488_=7.997; *P=*.005), time by transgender identity interaction was not a significant predictor after controlling for the intake PHQ score (*F*_1,488_=1.82; *P=*.18). Transgender patients reported significantly more depression at intake, but changes in depression scores were equivalent over time, regardless of transgender identity. The model run testing the relationship between gender and change in depressive symptoms indicated a significant improvement in PHQ scores over time (*F*_1,488_=7139.85; *P<*.001; partial η^2^=0.31), but gender was not significant in the model (*F*_1,488_=1.11; *P=*.85). Thus, the aggregate findings suggest that there was no difference in the change over time in depression by gender and transgender identity.

##### Depression Change Between Sexual Orientation Subgroups

A repeated measures ANOVA testing the relationship between identifying as bisexual and change in depressive symptoms indicated a significant change in PHQ scores over time (*F*_1,489_=240.25; *P<*.001). The time by bisexual identity interaction was significant (*F*_1,489_=4.67; *P=*.03); however, this finding was not considered statistically significant, given a Bonferroni correction of ⍺=.0125. On average, bisexual patients reported a reduction of 6.73 points on the PHQ from intake to discharge, whereas nonbisexual patients reported a reduction of 5.09 points on the PHQ. The model testing the relationship between identifying as pansexual and change in depressive symptoms from intake to discharge indicated a significant change in PHQ scores over time (*F*_1,489_=180.69; *P<*.001). The time by pansexual identity interaction was not significant (*F*_1,489_=0.90; *P=*.34). On average, pansexual patients reported a reduction of 5.10 points on the PHQ from intake to discharge, whereas nonpansexual patients reported a reduction of 5.88 points on the PHQ.

#### Suicide Risk

Across the sample of LGBTQIA+ patients who screened positive for passive suicidal thoughts at intake (295/551, 53.5%), 50.5% (149/295) no longer screened positive for passive suicidal thoughts at discharge (*P<*.001). Similarly, a significant change was detected for active suicidal ideation, wherein 72.1% (160/222) of the patients who screened positive at intake screened negative at discharge (*P<*.001).

##### Passive Suicidal Ideation Change Between Gender Subgroups

The logistic regression model (*χ*^2^_2_=56.1, *P<*.001; Nagelkerke *R*^2^=0.16), including transgender identity and passive SI intake, significantly predicted passive SI at discharge. After controlling for passive SI intake, transgender identity was not a significant predictor of passive SI at discharge (*P*=.17; 95% CI 0.88-2.16). Similarly, the overall regression model test containing passive SI at intake and gender identity significantly predicted passive SI at discharge (*χ*^2^_2_=55.8, *P<*.001; Nagelkerke *R*^2^=0.17); however, gender was not a significant predictor of passive SI at discharge (*P*=.83; 95% CI 0.73-1.29).

##### Passive Suicidal Ideation Change Between Sexual Orientation Subgroups

The overall logistic regression model containing passive SI at intake and bisexual orientation was significant (*χ*^2^_2_=60.2, *P<*.001; Nagelkerke *R*^2^=0.18). After controlling for passive SI at intake, nonbisexual clients were 1.64 times more likely to report passive SI at discharge relative to bisexual patients (*P*=.03; odds ratio [OR] 0.61, 95% CI 0.39-0.95), yet this finding was not considered statistically significant, given a Bonferroni correction of ⍺=.0125. Similarly, the overall model containing passive SI at intake and pansexual orientation was significant (*χ*^2^_2_=55.4, *P<*.001; Nagelkerke *R*^2^=0.16). However, after controlling for passive SI at intake, pansexual clients were no longer likely to report passive SI discharge (*P*=.76; 95% CI 0.68-1.69).

##### Active Suicidal Ideation Change Between Gender Subgroups

The logistic regression model containing transgender identity and active SI at intake significantly predicted active SI at discharge (*χ*^2^_2_=30.1, *P<*.001; Nagelkerke *R*^2^=0.10). After controlling for active SI at intake, transgender clients were still 1.8 times more likely to report active SI at discharge (*P*=.02; OR 1.84, 95% CI 1.12-3.03); however, this finding was not considered statistically significant given a Bonferroni correction of ⍺=.0125. Although the model containing gender identity and active SI at intake was significant (*χ*^2^_2_=29.6, *P<*.001; Nagelkerke *R*^2^=0.10), gender was not a significant predictor of active SI at discharge (*P*=.05; 95% CI 0.52-1.00).

##### Active Suicidal Ideation Change Between Sexual Orientation Subgroups

The logistic regression model containing active SI at intake and bisexual orientation was significant (*χ*^2^_2_=26.4, *P<*.001; Nagelkerke *R*^2^=0.09). After controlling for active SI at intake, nonbisexual clients were not significantly more likely to report active SI at discharge than bisexual patients (*P*=.35; 95% CI 0.47-1.30). Similarly, the overall model containing active SI at intake and pansexual orientation was significant (*χ*^2^_2_=27.5, *P<*.001; Nagelkerke *R*^2^=0.09). However, after controlling for active SI at intake, pansexual clients were no longer likely to report active SI at discharge (*P*=.15; 95% CI 0.87-2.40).

#### Clinical NSSI

The findings of the McNemar test indicated significant improvement in clinical NSSI, such that 55.1% (109/198) of patients who met the criteria at intake no longer met the criteria for clinical NSSI at discharge (*P<*.001).

##### NSSI Change by Gender Subgroups

The overall regression model containing NSSI at intake and transgender identity was significant (*χ*^2^_2_=35.4, *P<*.001; Nagelkerke *R*^2^=0.13). After controlling for NSSI criteria, transgender clients were 2 times as likely to report clinical NSSI at discharge (*P*=.01; OR 2.04, 95% CI 1.24-3.34). The regression model (*χ*^2^_2_=28.4, *P<*.001; Nagelkerke *R*^2^=0.10), containing gender identity and NSSI at intake, significantly predicted NSSI at discharge (*P*<.001; 95% CI 2.04-5.25); however, gender was not a significant predictor (*P*=.23; 95% CI 0.89-1.65).

##### NSSI Change by Sexual Orientation Subgroups

The overall logistic regression model containing NSSI at intake and bisexual orientation was significant (*χ*^2^_2_=27.0, *P<*.001; Nagelkerke *R*^2^=0.10). After controlling for meeting the NSSI criteria at intake, bisexual clients were no longer likely to report clinical NSSI at discharge (*P*=.93; 95% CI 0.61-1.58). Similarly, the overall model containing NSSI at intake and pansexual orientation was significant (*χ*^2^_2_=27.5, *P<*.001; Nagelkerke *R*^2^=0.10). After controlling for meeting the NSSI criteria at intake, pansexual clients were no longer likely to report clinical NSSI at discharge (*P*=.46; 95% CI 0.74-1.95).

## Discussion

### Principal Findings

This evaluation included a large sample of youth who self-identified as part of the LGBTQIA+ community to assess clinical acuity, treatment outcomes, and differences by subgroups. Similar to population research on the disproportionate rates of clinical presentation and severity among LGBTQIA+ youth relative to non-LGBTQIA youth, this study found statistically significant differences in clinical intake scores, indicating heightened clinical acuity within the LGBTQIA+ community. At treatment entry, LGBTQIA+ patients were significantly more likely to report active suicidal ideation, passive suicidal ideation, history of suicide attempts, clinical NSSI, and significantly worse depression. Even within the LGBTQIA+ patient sample, transgender youth and youth who identified as pansexual were more likely to endorse NSSI than nontransgender and nonpansexual youth, respectively, indicating that some minority statuses within the LGBTQIA+ community place youth at even higher risk. This latter finding reflects population statistics on the increased risk of self-harm among transgender patients [63]. These elevated risks fit within minority stress theories, as increased prejudice and stigma lead to additional mental strain for transgender and nonbinary youth, with transgender youth currently facing great societal strains [64].

Although more severe at baseline, LGBTQIA+ patients demonstrated considerable clinical improvement in all outcomes from intake to discharge. Among LGBTQIA+ youth who met the criteria for depression at treatment entry, PHQ-A scores improved significantly from an average pretreatment score in the moderately severe range to an average posttreatment score in the moderate range. Similar improvements were observed across suicide risk and NSSI, with 72.1% (160/222) of LGBTQIA+ youth who endorsed active suicidal ideation at intake no longer reporting it at discharge and 55.1% (109/198) of patients who met the criteria for clinical NSSI no longer meeting the criteria at discharge. This study adds to the preliminary research documenting positive mental health outcomes following LGBTQIA+ programming and represents the first study, to our knowledge, to investigate the outcomes of a web-based IOP with LGBTQIA+ youth.

More than half (557/878, 63.4%) of the clients in this sample reported at least one LGBTQIA+ identity, which is a high proportion relative to the national population and to other IOPs. This may be because of services being provided on the web, as digital tools have high acceptability and retention among LGBTQIA+ clients or to publicizing LGBTQIA+ clinicians and adapted curricula, which are not widely available. Furthermore, there might be a snowball effect. Early research on digital interventions for sexual and gender minority youth suggests that these youth will be more engaged in treatment when they connect with other youth with similar characteristics [65]. It may be that LGBTQIA+ youth interact with other youth with similar characteristics during treatment and have a positive experience, leading them to recommend Charlie Health to other LGBTQIA+ youth.

Although LGBTQIA+ clients demonstrated equitable change across most outcomes, transgender clients were 2 times more likely to report clinical NSSI at discharge. This implies that even within an intentionally designed IOP curriculum, additional barriers to clinical improvement remain for transgender patients to achieve equitable outcomes. This disparity at discharge may be because of the ongoing stigma and strain faced by gender-diverse youth [64]. In a recent qualitative study, Tilley et al [9] conducted focus groups and interviews with 21 “transgender and gender-diverse” young adults (aged 18-25 years) and 10 mental health clinicians to better understand how to adapt DBT to this population of youth. The authors tentatively concluded that the DBT curriculum could be adapted to focus more on skills related to self-care and awareness (vs interpersonal effectiveness), recognizing that this population has a higher exposure to psychologically distressing victimization because of their gender identity. Such nuanced, patient-centered analyses are fundamental to the design and evaluation of population-specific programming.

### Strengths and Limitations

A considerable strength of the current evaluation is access to a large, high-acuity sample of youth and young adults who identify as LGBTQIA+ as well as data that allowed for nuanced responses within these categories, where youth could select as many categories of identification as they felt resonated with them. However, because this was a clinical sample of LGBTQIA+ youth and young adults in a specialized program, these findings may not generalize to LGBTQIA+ youth in more general, nonspecialized programs. For instance, this analysis only included patients who attended 6 or more intensive outpatient sessions, which introduces an unknown number of biases that make the results not generalizable to the entire population of patients in care at Charlie Health. Demographic information beyond age, gender, and sexual identity was not collected at pretest and thus was not available for this study. The discharge survey was initially determined to be the best time point to ascertain honest responses from youth and young adults on sensitive demographic questions without undue influence from the presence of parents or guardians as the questions were asked, but the unintended consequence was not obtaining these data at the population level. Demographic factors, such as race, ethnicity, region, socioeconomic status, and living situation, have implications for mental health and treatment and should be considered in future research.

This is a program evaluation study, and it was therefore not possible to compare clients to a control group or to a group participating in a curriculum without adaptations. The study lacks a methodology to conclusively determine whether youth improve because of the adapted program or whether time or a more general program could have led to the same outcomes. The outcome data collected are general and cannot measure LGBTQIA-specific outcomes that could identify the mechanisms of treatment effectiveness. Furthermore, this study relied on self-reported outcomes, and the analysis was restricted to binary outcomes in some clinical areas. Demographic information (ie, sexual orientation and gender) was obtained using single-response, multiple-choice options—limiting patients’ ability to select all options that may apply in terms of describing their gender identity. Finally, although this investigation focused on quantitative differences in clinical symptoms following treatment, future evaluations would benefit from the inclusion of qualitative feedback from LGBTQIA+ youth in care to better understand subgroup needs, including why youth who identify as transgender evince smaller improvements in all symptom categories.

### Conclusions

This program evaluation is the first to evaluate LGBTQIA+-specific programming for youth in remote IOP, finding that youth had significant reductions in depression, suicidality, and NSSI symptoms. General population research reflects what was discovered in this analysis: LGBTQIA+ youth enter treatment at a heightened risk for suicidality, depression, and self-harm. Charlie Health addresses this need by adapting its general curriculum to intentionally address issues salient to the LGBTQIA+ community. After participating in the program, most clients reported significant improvements in depression and substantial reductions in suicidal ideation and NSSI. Further research on identity-affirming and culturally competent care is necessary to ensure equitable outcomes for the most vulnerable youth.

## Abbreviations

ABASI: Alexian Brothers Assessment of Suicidal Ideation
ASQ: Ask Suicide-Screening Questionnaire
DBT: dialectical behavior therapy
IOP: intensive outpatient program
LGBTQIA+: lesbian, gay, bisexual, transgender, queer, intersex, asexual, and other minoritized gender and sexual identities
NSSI: nonsuicidal self-injury
OR: odds ratio
PHQ: Patient Health Questionnaire
PHQ-A: Patient Health Questionnaire for Adolescents
SI: self-injury

## Data Availability

Owing to the quality improvement nature of this study, participants did not agree to their data being publicly shared; therefore, supporting data were not available.
